# Supplementary material for: Bone Regeneration of Critical-Size Calvarial Defects in Rats Using Highly Pressed Nano-Apatite/Collagen Composites
Source: Materials (Basel). 2022 May 8;15(9):3376. doi: 10.3390/ma15093376 (PMC9099897; doi:10.3390/ma15093376)
Supplement: Supplementary file 1 [file materials-15-03376-s001.zip › materials-1713220-supplementary.pdf]

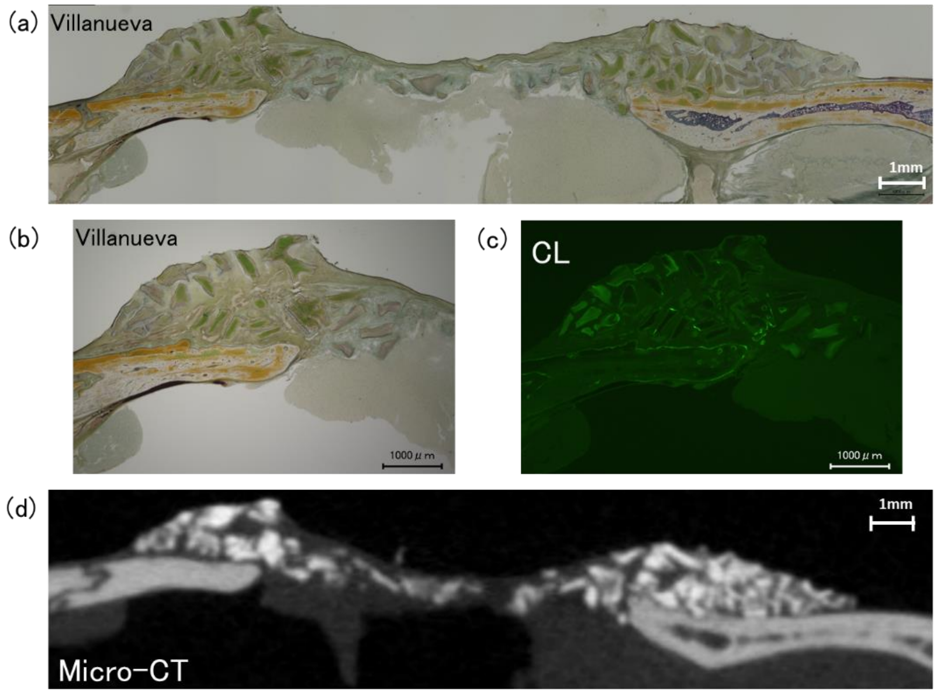

Figure S1: Villanueva-stained histological images of the zone obtained from the rat cranial bone defect filled with one commercial alloplast (Bio-Oss) at 8 weeks after surgery at low magnification (a) and high magnification (b) with CL- fluorescent labeling (c); micro-CT sagittal section image (d).
